# Understanding the Occurrence of Psychiatric Disorders in Epilepsy in Brazil: An Epidemiological Investigation

**DOI:** 10.1192/j.eurpsy.2024.243

**Published:** 2024-08-27

**Authors:** L. Bardini Goulart, A. Roloff Krüger, G. Moreno Xavier, G. Fiorio Grando, J. Michelon, L. F. Alves Nascimento, J. Adames, A. T. Konzen, G. Pereira Bernd, C. Fontes Augusto, H. Wolmeister, I. Fachinetto Thoen, Y. de França, P. H. Filipin Von Muhlen, F. J. Carvalho da Costa, V. Kayser, P. H. Paesi Dutra, R. Rahal de Albuquerque, T. Garcia Furtado, G. Macelaro, A. C. Castelo, H. Vieira Rodrigues, E. Rockenbach Fidélis, D. Crusius, E. Guidugli, M. F. Valentim de Paula, Y. Marques Loureiro, E. Paiva Borsa, L. de Paula e Souza, G. Ferreira Cruz

**Affiliations:** ^1^Pontifical Catholic University of Rio Grande do Sul, Porto Alegre; ^2^Lutheran University of Brazil, Canoas; ^3^Federal University of Health Sciences of Porto Alegre; ^4^Federal University of Rio Grande do Sul, Porto Alegre, Brazil

## Abstract

**Introduction:**

Epilepsy is one of the most common serious brain illness, with symptoms influenced by multiple risk factors and a strong genetic predisposition, rather than having a single expression and cause¹. Neuropsychiatric symptoms in epilepsy can encompass manifestations such as mood alterations, anxiety, sleep disturbances, psychosis, and behavioral disorders. While the motor and sensory manifestations of epileptic seizures are widely recognized, neuropsychiatric symptoms accompanying epilepsy are often underestimated. Therefore, it is essential to understand the most prevalent epidemiological profile of these patients to improve the diagnosis and management of these symptoms.

**Objectives:**

Our goal was to evaluate the neuropsychiatric behavior of epilepsy patients in Brazilian over the past 3 years through hospitalization data in order to outline an epidemiological and behavioral profile.

**Methods:**

A cross-sectional, descriptive, retrospective, and quantitative study was conducted on hospitalizations of individuals simultaneously diagnosed with epilepsy, schizotypal and delusional disorders, and mood disorders in all five regions of Brazil (South, Southeast, Midwest, North, and Northeast) between February 2020 and December 2022. Data from January 2020 were not available. The data used were collected through the Department of Health Informatics of the Brazilian Unified Health System (DATASUS) in the “Hospital Information System of SUS” section, gathering information regarding the nature of care, age range, gender, and ethnicity of the patients.

**Results:**

The analysis covers the years 2020 to 2022, totaling 503,045 hospitalizations. In 2022, the highest number of cases occurred (≈ 37.55%), followed by 2021 (≈ 33.62%) and 2020 (≈ 28.81%). Urgent hospitalizations represented ≈ 90.85% of the total. The most affected age group was 30 to 39 years old (≈ 18.30%). Men were more affected than women (≈ 52.03% and ≈ 47.96%, respectively), and Caucasians accounted for ≈ 36.07% of the hospitalizations. The average length of stay was 19.1 days, and the mortality rate was 1.4%.

**Conclusions:**

Thus, there is a gradual and annual increase in the number of hospitalizations during the observed period. While there is a minimal disparity between the affected genders, it is evident that the profile of male, caucasian, and adult patients is the most prevalent. Moreover, the predominantly urgent nature of hospitalizations points to an alarming scenario regarding this issue. From the analysis of the data obtained in the study, there is a clear need for interventions capable of reducing the prevalence of hospitalizations for neuropsychiatric symptoms in epilepsy patients in Brazil.

**Disclosure of Interest:**

None Declared

